# Supplementary material for: Evidence supported by Mendelian randomization: impact on inflammatory factors in knee osteoarthritis
Source: Front Med (Lausanne). 2024 May 28;11:1382836. doi: 10.3389/fmed.2024.1382836 (PMC11165061; doi:10.3389/fmed.2024.1382836)
Supplement: Supplementary file 1 [file Data_Sheet_1.ZIP › data/GCST90274846_5e6/again/846.forest.pdf]

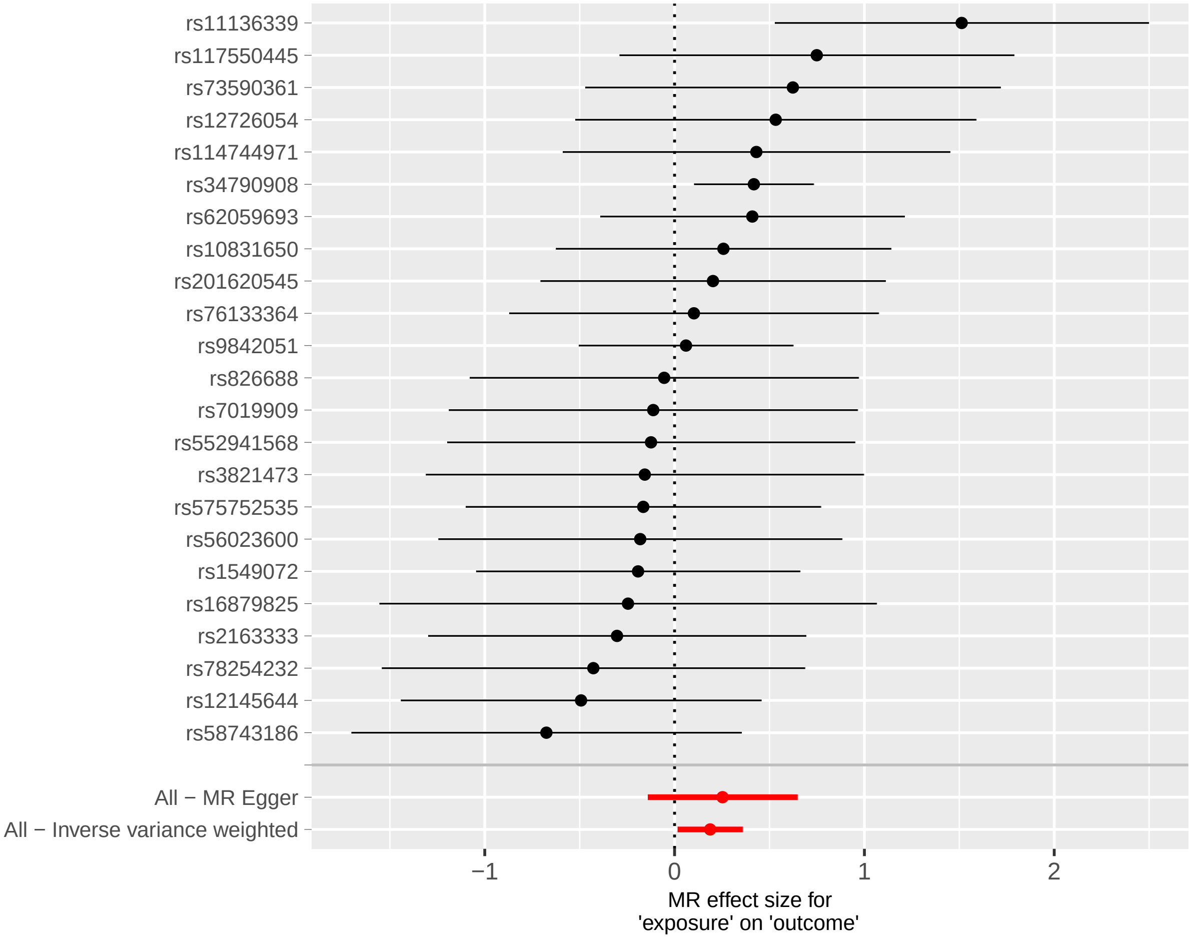
rs11136339
rs117550445
rs73590361
rs12726054
rs114744971
rs34790908
rs62059693
rs10831650
rs201620545
rs76133364
rs9842051
rs826688
rs7019909
rs552941568
rs3821473
rs575752535
rs56023600
rs1549072
rs16879825
rs2163333
rs78254232
rs12145644
rs58743186
All − MR Egger
All − Inverse variance weighted
−1
0
1
2
MR effect size for
'exposure' on 'outcome'
